# Supplementary material for: Additively-Manufactured High-Concentration Nanocellulose Composites: Structure and Mechanical Properties
Source: Polymers (Basel). 2023 Jan 28;15(3):669. doi: 10.3390/polym15030669 (PMC9920716; doi:10.3390/polym15030669)
Supplement: Supplementary file 1 [file polymers-15-00669-s001.zip › polymers-2162399-supplementary.pdf]

Supplementary Materials

Additively-Manufactured High-Concentration Nanocellulose Composites: Structure and Mechanical Properties

Muhammad Latif, Yangxiaozhe Jiang, Jongmin Song and Jaehwan Kim *

Creative Research Center for Nanocellulose Future Composites, Department of Mechanical Engineering, Inha University, 100, Inha-ro, Michuhol-gu, Incheon 22212, Republic of Korea
* Correspondence: jaehwan@inha.ac.kr

Table S1. The composition of all prepared PVA-blended high-concentration NC pastes.

Samples	CNF (g)	CNC (g)	PVA		Mixing ratio CNC: CNF: PVA
			wt%	weight (g)	
CNC-CNF	1.22	24.23	-	-	20:1:0
10PVA-NC	1.22	24.23	10	2.4	20:1:1
15PVA-NC	1.22	24.23	15	3.63	20:1:1
20PVA-NC	1.22	24.23	20	4.84	20:1:1

Table S2. Optimized printing parameters for high-concentration NC paste.

Paste	Syringe (ml)	Needle diameter (mm)	Feeding rate (ml/min)	Printing speed (mm/s)	Screw speed (rpm)
CNC-CNF	60	1.51	1.2	9.37	150

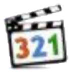

Extrudability (NC-PVA).avi

Video S1. Extrudability of the prepared 15 wt% PVA-mixed NC paste and freestanding of wet structures in a layer-wise pattern.

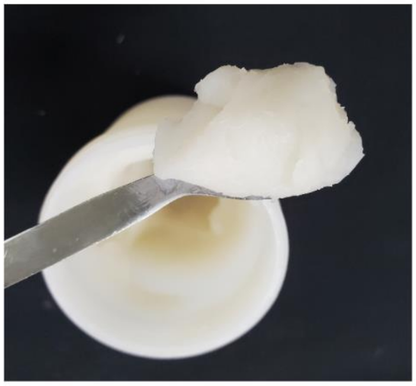

Figure S1. The 15 wt% PVA-mixed nanocellulose paste contains the highest possible nanocellulose (25.73 wt%).

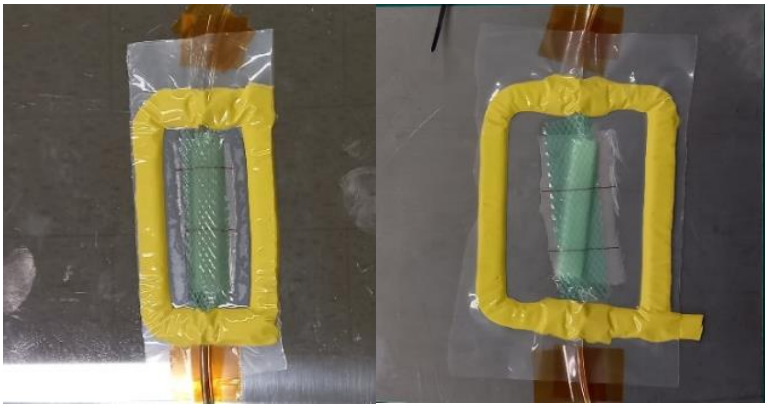

Figure S2. Infusion of Epofix resin to the freeze-dried beam-shaped samples printed with PVA-blended NC paste via the VARTM method.

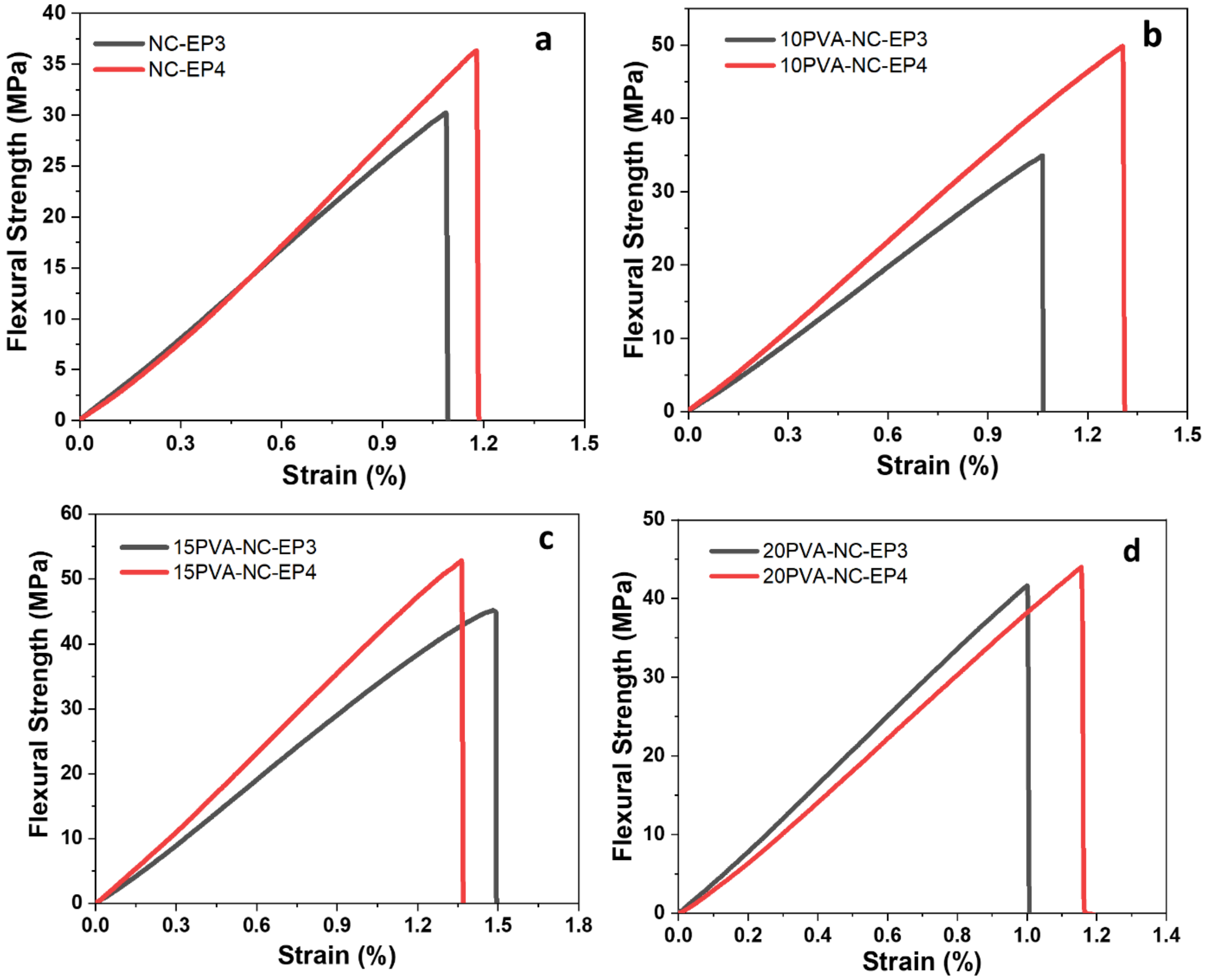

Figure S3. Stress–strain curves of (a) Pristine, (b) 10 wt% PVA-NC, (c) 15 wt% PVA-NC, and (d) 20 wt% PVA-mixed NC 3D-printed specimens followed by freeze-drying and resin infusions.

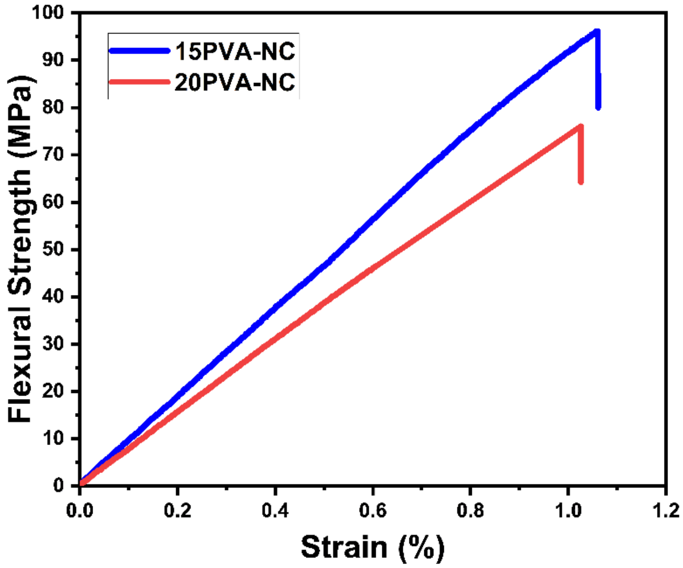

Figure S4. Stress–strain curves of PVA-NC paste samples dried in cleanroom conditions.
